# Can Antinuclear Antibodies (ANA) be Monoclonal?

**DOI:** 10.1155/2021/7006466

**Published:** 2021-09-30

**Authors:** Laura Biederman, Anjali A. Satoskar, Mohankumar Doraiswamy, Samir Parikh, Brad Rovin, Brian Mussio, Galina Mikhalina, Sergey V. Brodsky

**Affiliations:** ^1^Department of Pathology, The Ohio State University Wexner Medical Center, Columbus, OH, USA; ^2^Department of Pathology, Nationwide Children's Hospital, Columbus, OH, USA; ^3^Department of Medicine, The Ohio State University Wexner Medical Center, Columbus, OH, USA; ^4^Department of Pulmonary and Critical Care Medicine, Texas Tech University Health Science Center, Lubbock, TX, USA; ^5^Hypertension Nephrology Consultants Inc., Columbus, OH, USA; ^6^Rochester Regional Health Nephrology, Rochester, NY, USA

## Abstract

**Background:**

Nuclear staining by immunofluorescence in a kidney biopsy is often seen in patients with positive antinuclear antibodies (ANA) in the serum. These ANA are usually polyclonal, but herein we report 9 cases with an unusual finding of monoclonal nuclear staining by immunofluorescence on kidney biopsy. *Case Presentation*. Nine cases with predominant stain for kappa or lambda light chain were identified by searching the renal pathology laboratory database for the past 10 years. All cases had positive stain for only kappa (six cases) or lambda (three cases) light chain in the nuclei. Eight out of nine cases had positive nuclear IgG stain, and one case had positive nuclear IgA stain. Among cases with positive nuclear IgG staining, six cases were positive for IgG1 subclass, one case was positive for IgG2 subclass, and one case was positive for IgG3 subclass. All patients with positive IgG nuclear stain, who had testing for ANA, had positive ANA. Patients with positive IgG1 subclass did not have monoclonal protein in the serum or urine, but the patient with positive IgG2 subclass and lambda light chain stain in the nuclei had IgG lambda monoclonal gammopathy.

**Conclusions:**

We identified a new unique pattern of nuclear stain by immunofluorescence in kidney biopsies that suggests the presence of monoclonal ANA. Workup for underlying monoclonal gammopathy is warranted in such patients.

## 1. Introduction

Antinuclear antibodies (ANA) are autoantibodies that are often present in patients with autoimmune diseases, such as systemic lupus erythematous (SLE), Sjogren's syndrome, scleroderma, mixed connective tissue disease, polymyositis, and dermatomyositis. The first method to identify ANA is considered one of the milestones in the history of clinical immunology over the last 60 years [1]. ANA is a screening test to detect autoimmune antibodies, and, if positive, other tests to detect specific markers such as anti-dsDNA antibodies or antiextractable nuclear antigens (anti-ENA) antibodies (such as SS-A, SS-B, Sm, Sm/RNP, Jo-1, and Scl-70) are performed [1]. The traditional methods for detecting ANA are indirect immunofluorescence (IIF) and enzyme-linked immunosorbent assay (ELISA). Renal pathologists use immunofluorescence to detect deposition of different immunoglobulins and other proteins in the kidney. If in a kidney biopsy there is a positive nuclear stain for an immunoglobulin, usually IgG, by immunofluorescence, this indicates the presence of ANA in the patient. In addition to positive IgG, positive nuclear stain is usually seen for both kappa and lambda light chains [2]. Herein, we report nine cases of positive nuclear stain by immunofluorescence in kidney biopsies that show the presence of monoclonal ANA.

## 2. Case Presentation

Renal and transplant pathology laboratory database at the Ohio State University Wexner Medical Center (OSUWMC) between January 1, 2010, and June 30, 2021, was searched for terms “nuclear staining” in the section “immunofluorescence findings.” Each individual report was analyzed, and nine cases with positive nuclear staining for only kappa or lambda light chain were selected for studies. Direct immunofluorescence with antibodies to IgG subclasses was performed in cases with positive nuclear staining for IgG (eight cases). Clinical history and laboratory data were analyzed for the presence of a monoclonal protein in the serum and urine.

Demographic and laboratory data for patients are present in Table 1. There were 6 females and 3 males, and all patients were Caucasian. The mean age was 58 ± 24 years (range 22–84 years). Three out of 9 patients had acute kidney injury at the time of the kidney biopsy. Three patients had nephrotic range proteinuria and two had severe hematuria. Seven patients had positive ANA in the serum, one case had negative ANA in the serum, and one case did not have ANA data available. Monoclonal gammopathy workup was performed in 5 of the 9 patients. Two of these 5 patients(cases # 1 and 7) did not have monoclonal protein in the serum and/or urine. Three patients had abnormalities detected on protein electrophoresis or immunofixation (Table 1). Bone marrow biopsies were performed in two out of these three patients and both were negative for multiple myeloma.

**Figure 1:**
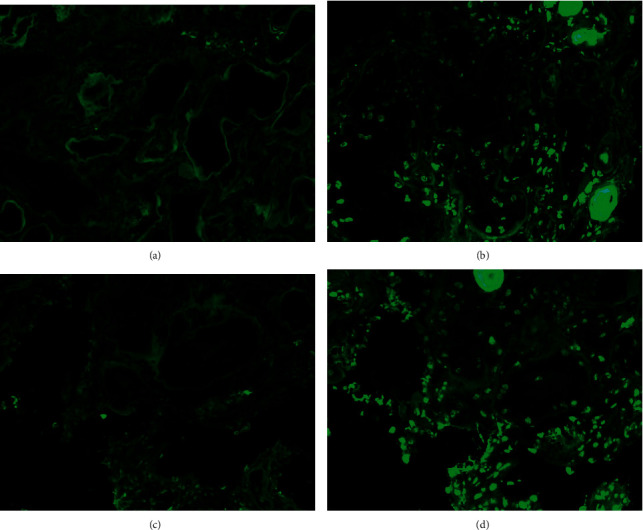
Immunofluorescence findings in a patient with positive IgA nuclear staining. (a) Negative stain for IgG in the nuclei. (b) Positive stain for IgA in the nuclei. (c) Negative stain for kappa light chain in the nuclei. (d) Positive stain for lambda light chain in the nuclei. Magnification 200x.

**Figure 2:**
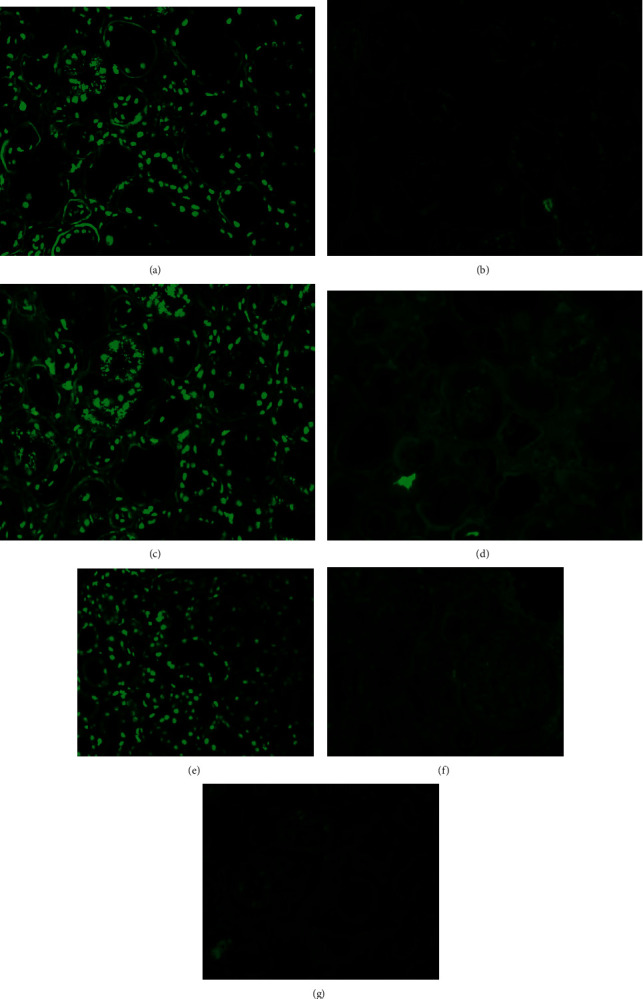
Immunofluorescence findings in a patient with IgG lambda monoclonal gammopathy. (a) Positive stain for IgG in the nuclei. (b) Negative stain for kappa light chain in the nuclei. (c) Positive stain for lambda light chain in the nuclei. (d) Negative stain for IgG1 subclass in the nuclei. (e) Positive stain for IgG2 subclass in the nuclei. (f) Negative stain for IgG3 subclass in the nuclei. (g) Negative stain for IgG4 subclass in the nuclei. Magnification 200x.

Immunofluorescence findings in kidney biopsies are present in Table 2. There was positive nuclear stain for IgG in all of the cases except case #6, where IgA nuclear staining was seen (Figures 1(a)–1(c)). There was predominant staining for one of the light chains in all 9 cases, with 6 (66.7%) cases having predominance of kappa light chain and 3 (33.3%) cases with predominant lambda light chain nuclear stain. Among the 8 cases with positive IgG nuclear stain, 6 had dominant IgG1 subclass stain, one case (#4) had dominant IgG2 subclass (Figures 2(d)–2(g)), and one case (#8) had dominant IgG3 subclass stain (Table 2).

Main pathologic findings in the kidney biopsies are present in Table 3. Four patients had immune complex-mediated glomerulonephritis (three cases of lupus nephritis and one with predominant C3-containing immune complex deposits), and five patients did not have immune complex depositions in the glomeruli.

## 3. Discussion and Conclusions

To the best of our knowledge, this is the first report describing the monoclonal pattern of staining of tissue ANA in kidney biopsies. ANA are an important diagnostic marker in the variety of autoimmune diseases that have predictive and prognostic values [3]. ANA are a heterogeneous group that include several different antibodies against specific components of the nucleus and nuclear envelope [1]. We identified 9 cases where there was strong predominance for kappa or lambda light chain stain in the nuclei by immunofluorescence. Nuclear stain in a kidney biopsy indicates presence of ANA in the patient. Indeed, 7 out of 9 patients in this study had positive ANA, and only one patient had negative ANA testing, but this patient had positive staining for IgA, not IgG, in the nuclei. This patient underwent testing by using the Euroimmun immunofluorescence assay (IFA) for ANA screening. This methodology uses goat anti-human IgG-conjugated antibodies and does not recognize IgA [4]. Therefore, this patient may still have ANA in the serum which are not detectable by this testing method.

In 8 patients with positive IgG nuclear stain, 6 cases had predominant stain for IgG1 subclass. While IgG1 is being the predominant subclass in the circulation, these data should be interpreted with caution. However, the predominance of IgG1 should also not discount these data entirely. As it was illustrated in the case series that describe proliferative glomerulonephritis with monoclonal IgG deposits, 9 out of 32 patients also had predominance of IgG1 subclass, and in these patients, there was evidence of IgG1-containing immune complex deposits on a renal biopsy, indicating that this IgG1 was pathogenic [5]. Interestingly, in our cases and in those described by Nasr et al., monoclonal protein in the serum or urine usually was not found in patients who had predominance of the IgG1 subclass [5]. Case #4 showed specificity for IgG2 subclass and lambda light chain in the nuclei, and this patient had monoclonal IgG lambda in the serum. This is notable because this is the only patient with the monoclonal paraprotein in the serum that corresponds to the kidney biopsy immunofluorescence findings.

Case #4 makes a compelling argument that monoclonal ANA stain can be caused by a paraprotein in a subset of patients. Based on these results, another subset of patients may have a detectable paraprotein that does not correspond to the ANA stain on immunofluorescence. The differential diagnosis includes monoclonal gammopathy with very low levels of monoclonal protein that is below the current methodology detection limits [6]. Dreher in 1977 described that in patients with rheumatoid arthritis, the majority of ANA are IgG ANA (43%), followed by IgM ANA (20%) with as little as 2% of IgA ANA [7]. Our data also indicate that this trend largely holds for monoclonal ANA as well. In our study, the majority are IgG ANA (8 out of 9 cases, 89%) with one case (11%) of IgA ANA. Several of our patients had diagnosis of SLE. In patients with lupus, it is not uncommon to see nuclear ANA stain by immunofluorescence, though it is nearly always polyclonal. The significance of the monoclonal ANA stain in these patients is unclear. SLE and other lupus-like inflammatory conditions [8, 9] are associated with an increased malignancy risk, especially lymphomas. Although this monoclonal nuclear stain may not indicate a paraprotein, work up for neoplastic processes may be prudent in these patients event though polyclonal nuclear stain is relatively common.

In summary, we identified the unusual pattern of stain in the nuclei that suggests the presence of a monoclonal ANA. Even though only one patient had the corresponding monoclonal protein in the serum, several patients showed different monoclonal paraprotein on additional work up. Therefore, our data suggest that the presence of the monoclonal pattern of nuclear stain in a kidney biopsy warrants clinical workup for monoclonal gammopathy.

## Figures and Tables

**Table 1:** Demographic and laboratory data of the participants.

Case	Age	Gender	Race	BL SCr (mg/dl)	SCr at the time of bx (mg/dl)	Proteinuria	Hematuria	ANA	Monoclonal gammopathy
1	84	F	C	1.1	3.6	n/a	TNTC	1 : 640	SPEP/UPEP negative
2	69	F	C	2.3	2.8	0.6 g/g	100/HPF	1 : 640	Not performed
3	22	F	C	1.1	1.1	0.5 g/g	1–5/HPF	1 : 320	Not performed
4	72	M	C	1.0	3.1	0.4 g/g	n/a	1 : 320	Monoclonal IgG lambda, serum
5	36	F	C	n/a	2.0	1.9 g/g	6–10/HPF	1 : 640	Not performed
6	77	F	C	2.0	2.2	6 g/g	n/a	Neg	Polyclonal IgA, serum,
7	23	F	C	0.86	0.86	4.5 g/24 h	6–9/HPF	Positive	Serum IF negative
8	77	M	C	1	1	7 g/g	n/a	Positive	Monoclonal IgM kappa, serum
9	62	M	C	1.8	1.8	n/a	n/a	n/a	Not performed

**Table 2:** Nuclear staining pattern by immunofluorescence.

Case #	IgG1	IgG2	IgG3	IgG4	Kappa	Lambda	Others
1	1+	0	0	0	1+	0	
2	1+	0	0	0	1+	0	
3	1+	0	0	0	1+	0	
4	0	1+	0	0	0	1+	
5	2+	±	0	0	2+	±	
6					±	2+	IgA 2 + *n*
7	2+	0	0	±	2+	±	
8	0	0	1+	0	0	1+	
9	2+	0	0	0	2+	0	

Intensity of the staining was quantitated by a semiquantitative scale, where 0 represents absent; 1+ represents mild; 2+ represents moderate, and 3+ represents prominent staining. BL, baseline; SCr, serum creatinine; ANA, antinuclear antibody; SPEP/UPEP, serum and urine protein electrophoresis.

**Table 3:** Main histologic findings in the kidney biopsies.

Case #	# of glomeruli	# of globally sclerosed glomeruli	IFTA	Pathologic diagnosis
1	15	7	Prominent	Active focal crescentic and necrotizing glomerulonephritis
2	8	0	Mild	Mild diffuse proliferative glomerulonephritis with “full-house” glomerular immune complex deposition.
3	69	28	Moderate	Focal crescentic glomerulonephritis with fibrous crescents and predominantly C3-containing mesangial immune complex deposits in the glomeruli
4	18	10	Moderate	Moderate chronic kidney injury with increased number of globally sclerosed glomeruli
5	3	1	Moderate	Focal proliferative lupus nephritis (ISN/RPS class III (A/C))
6	5	1	Moderate	Small amount of renal cortex with nonspecific findings
7	7	0	Moderate	Focal proliferative lupus nephritis with membranous lupus nephritis (ISN/RPS class III + V)
8	8	1	Mild	Membranous glomerulonephritis
9	17	6	Prominent	Advanced stage diabetic nephropathy with diffuse and nodular diabetic glomerulosclerosis.

## Data Availability

The data and pathology images used to support the findings of this study are available from the corresponding author upon request that will not violate HIPA rules about privacy of patient information.

## References

[1] Pisetsky D. S. (2017). Antinuclear antibody testing-misunderstood or misbegotten?. *Nature Reviews Rheumatology*.

[2] McCoy R. C. (1972). Nuclear localization of immunoglobulins in renal biopsies of patients with lupus nephritis. *American Journal Of Pathology*.

[3] Milkiewicz M., Caballería L., Smyk D. S., Milkiewicz P. (2012). Predicting and preventing autoimmunity: the case of anti-mitochondrial antibodies. *Autoimmunity Highlights*.

[4] Ricchiuti V., Adams J., Hardy D. J., Katayev A., Fleming J. K. (2018). Automated processing and evaluation of anti-nuclear antibody indirect immunofluorescence testing. *Frontiers in Immunology*.

[5] Nasr S. H., Satoskar A., Markowitz G. S. (2009). Proliferative glomerulonephritis with monoclonal IgG deposits. *Journal of the American Society of Nephrology*.

[6] Roth R. M., Benson D., Hebert L. A. (2013). Progressive renal light chain amyloidosis with the absence of detectable free monoclonal light chains after an autologous hematopoietic stem cell transplant for amyloid light chain amyloidosis. *Archives of Pathology & Laboratory Medicine*.

[7] Dreher R. (1977). [Heterogeneity of antinuclear antibodies (ANA), immunoglobulin- and complement levels in sera of patients with rheumatoid arthritis (RA) (author’s transl)]. *Immunitset und Infektion*.

[8] Nived O., Bengtsson A., Jönsen A., Sturfelt G., Olsson H. (2001). Malignancies during follow-up in an epidemiologically defined systemic lupus erythematosus inception cohort in southern Sweden. *Lupus*.

[9] Abu-Shakra M., Gladman D. D., Urowitz M. B. (1996). Malignancy in systemic lupus erythematosus. *Arthritis & Rheumatism*.

